# *MSN2* and *MSN4* Link Calorie Restriction and TOR to Sirtuin-Mediated Lifespan Extension in *Saccharomyces cerevisiae*

**DOI:** 10.1371/journal.pbio.0050261

**Published:** 2007-10-02

**Authors:** Oliver Medvedik, Dudley W Lamming, Keyman D Kim, David A Sinclair

**Affiliations:** Paul F. Glenn Laboratories for the Biological Mechanisms of Aging, Department of Pathology, Harvard Medical School, Boston, Massachusetts, United States of America; The Salk Institute, United States of America

## Abstract

Calorie restriction (CR) robustly extends the lifespan of numerous species. In the yeast *Saccharomyces cerevisiae,* CR has been proposed to extend lifespan by boosting the activity of sirtuin deacetylases, thereby suppressing the formation of toxic repetitive ribosomal DNA (rDNA) circles. An alternative theory is that CR works by suppressing the TOR (target of rapamycin) signaling pathway, which extends lifespan via mechanisms that are unknown but thought to be independent of sirtuins. Here we show that TOR inhibition extends lifespan by the same mechanism as CR: by increasing Sir2p activity and stabilizing the rDNA locus. Further, we show that rDNA stabilization and lifespan extension by both CR and TOR signaling is due to the relocalization of the transcription factors Msn2p and Msn4p from the cytoplasm to the nucleus, where they increase expression of the nicotinamidase gene *PNC1*. These findings suggest that TOR and sirtuins may be part of the same longevity pathway in higher organisms, and that they may promote genomic stability during aging.

## Author Summary

There are only a few techniques that reliably promote longevity in multiple, distantly related species. Perhaps the best known, caloric restriction (CR), was first shown to promote lifespan in rodents in the 1930s and has since been shown to work in most species it has been tested on. We and others have previously proposed that CR extends lifespan in budding yeast by boosting the activity of sirtuin deacetylases, which work to extend lifespan by suppressing genomic instability. A competing theory is that CR works by suppressing the TOR (target of rapamycin) signaling pathway, which has recently been discovered to extend the lifespan of yeast and worms, but the downstream players are not yet known. We show that TOR inhibition and sirtuins are part of the same CR pathway that extends yeast lifespan by stabilizing the genome. CR and TOR inhibition promote longevity by relocalizing two transcription factors, Msn2p and Msn4p, from the cytoplasm to the nucleus, where they increase expression of the nicotinamidase gene *PNC1,* a regulator of sirtuin activity. We propose that TOR signaling and sirtuins may also be part of the same CR pathway in mammals.

## Abstract 3

Both caloric restriction and suppression of the TOR signaling pathway are known to extend lifespan in yeast. Here, both manipulations are shown to act via the same mechanism.

## Introduction

In the budding yeast *Saccharomyces cerevisiae,* replicative lifespan is measured by the number of divisions that a mother cell undergoes before senescing [1–3]. A primary cause of aging in this organism is homologous recombination between ribosomal DNA (rDNA) repeats, resulting in the formation of extrachromosomal rDNA circles (ERCs) that accumulate to toxic levels in mother cells [4]. Sir2p and a closely related homolog, Hst2p, belong to the sirtuin family of NAD^+^-dependent deacetylases [5] that can forestall aging by stabilizing the rDNA locus [4,6,7]. Although rDNA recombination is not known to play a role in the aging of metazoans, the function of Sir2p enzymes in lifespan determination appears to be conserved. In Caenorhabditis elegans and *Drosophila melanogaster,* additional copies of the *SIR2* gene or pharmacological modulation of the Sir2p deacetylase also extend lifespan [8–11].

The diet known as calorie restriction (CR) prolongs the lifespan of numerous species, including fungi, invertebrates, and mammals [1,12,13]. Whether or not Sir2p enzymes play a role in CR-mediated lifespan extension is hotly debated. In support of their playing a role, additional copies of either *SIR2* or *HST2* suppress rDNA recombination and extend yeast replicative lifespan, whereas strains lacking *SIR2* and *HST2* fail to live longer when subjected to CR [14,15]. Similarly, CR diets or genetic mimics of CR fail to extend the lifespan of D. melanogaster and C. elegans lacking Sir2p [12,16]. However, other researchers favor a model in which Sir2p plays no role in CR-mediated lifespan extension, and instead the TOR (target of rapamycin) pathway is proposed to play the central role [17,18].

TOR is a nutrient-responsive phosphatidylinositol-kinase-related kinase that regulates protein synthesis and cell growth, and is inhibited by rapamycin, an immunosuppressive and anticancer drug that specifically inhibits TOR [19]. It has recently been discovered that lifespan can be extended in a variety of species by inhibition of TOR signaling, including *S. cerevisiae, C. elegans,* and D. melanogaster [17,20,21]. The mechanism by which inhibition of TOR signaling extends lifespan is unclear, but it has been proposed that it may act by altering ribosome assembly and translation [17,21–23]. Similar to CR, inhibition of TOR signaling can extend yeast lifespan in the absence of *SIR2* [17], but whether *SIR2* plays a role in lifespan extension during inhibition of TOR signaling is not known.

We have previously investigated the pathways by which CR operates in yeast. The enzymatic activity of Sir2p is regulated by endogenous levels of nicotinamide (NAM), a sirtuin inhibitor [24]. Yeast strains grown on standard 2% glucose medium have an intracellular concentration of ∼50 μM NAM, which is almost precisely the IC_50_ of Sir2p [24–28]. We and others have shown that CR and other mild stresses, including heat stress and osmotic shock, extend yeast lifespan by increasing expression of the *PNC1* gene, which encodes a nicotinamidase [27,29]. Recent evidence indicates that mammalian Nampt/PBEF, a putative functional ortholog of *PNC1* that is required for the conversion of NAM to NAD^+^, also regulates sirtuin activity [30,31].

*PNC1* is an intriguing longevity gene because its expression is regulated by environmental stimuli that extend lifespan, such as heat, osmotic stress, low amino acids, and CR. Binding sites for the stress-responsive zinc-finger transcription factors Msn2p and Msn4p have been identified in the *PNC1* promoter [32]. Msn2p and Msn4p have previously been shown to regulate chronological lifespan extension in response to deletion of the yeast Akt homolog *SCH9* by controlling the expression of the superoxide dismutase *SOD2* [33,34]. Msn2p/4p are therefore good candidates to regulate *PNC1* expression in response to nutrient availability, but a previous study, which used a *cdc25–10* mutant strain to mimic CR, concluded that *MSN2* and *MSN4* play no role in CR-mediated lifespan extension [14].

Here we show that Msn2p/4p are important regulators of yeast replicative lifespan and that they relocalize from the cytoplasm to the nucleus during CR, where they bind to and activate the *PNC1* gene. Moreover, inhibition of TOR signaling acts via this same pathway to promote the expression of *PNC1* and suppress rDNA recombination. These data provide evidence for a pathway from the cell's environment to an actual cause of aging, via which both CR and TOR signaling modulate lifespan.

## Results

**Figure 1:**
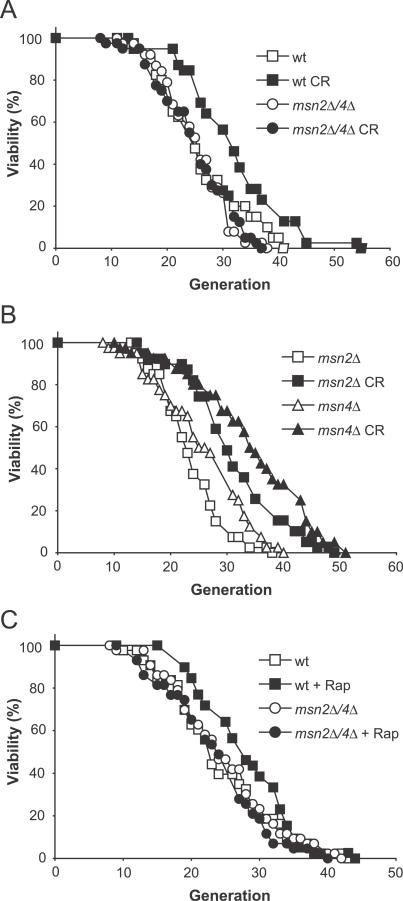
*MSN2/4* Are Required for Lifespan Extension by CR and Rapamycin (A) *MSN2/4* are required for lifespan extension by CR (growth on 0.5% glucose). Average lifespans: W303 (wild-type [wt]) on 2% glucose, 25.0 divisions; W303 on CR diet, 30.8; *msn2*Δ/*4*Δ on 2% glucose, 24.2; and *msn2*Δ/*4*Δ on CR diet, 23.9. (B) The lifespans of *msn2*Δ and *msn4*Δ single mutants can be extended by CR. Average lifespans: *msn2*Δ on 2% glucose, 22.5 divisions; *msn2*Δ on CR diet, 29.9; *msn4*Δ on 2% glucose, 24.7; and *msn4*Δ on CR diet, 33.2. (C) *MSN2/4* are required for lifespan extension by rapamycin (Rap). Average lifespans: wild-type, 23.3 divisions; wild-type + 1 nM rapamycin, 26.9; *msn2*Δ/*4*Δ, 23.8; and *msn2*Δ/*4*Δ + 1 nM rapamycin, 22.5.

We began by asking whether *MSN2/4* are mediators of yeast lifespan extension by CR (0.5% glucose). In contrast to previous work using a genetic mimic of CR [14], we found that lifespan extension by CR was completely *MSN2*/*4*-dependent (Figure 1A). Single deletions of *MSN2* or *MSN4* did not block the ability of CR to extend lifespan, consistent with their known redundancy (Figure 1B). Interestingly, *MSN2* and *MSN4* were also absolutely necessary for lifespan extension resulting from TOR inhibition (Figure 1C).

**Figure 2:**
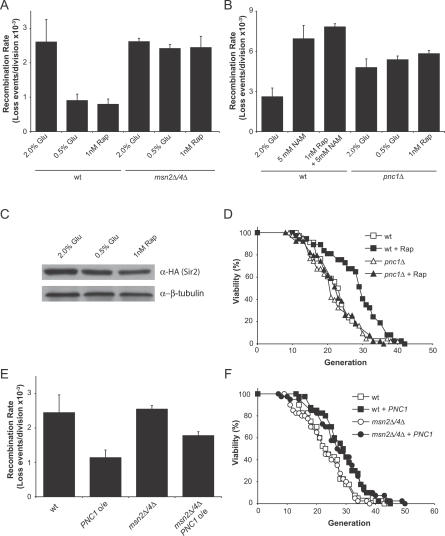
Effect of TOR Signaling on Sir2p Functions Requires *MSN2/4* and *PNC1* (A) Rapamycin and CR suppress rDNA recombination in an *MSN2/4*-dependent manner. (B) Deletion of *PNC1* or treatment with NAM blocks the ability of CR or rapamycin treatment to lower rDNA recombination. (C) CR and rapamycin treatment do not increase Sir2p levels. (D) Deletion of *PNC1* blocks the ability of rapamycin to extend lifespan. Average lifespans: wild-type (wt), 23.3 divisions; wild-type + 1 nM rapamycin, 26.9; *pnc1*Δ, 20.9; and *pnc1*Δ + 1 nM rapamycin, 21.4. (E) Overexpression (o/e) of *PNC1* suppresses rDNA recombination in both wild-type and *msn2*Δ/*4*Δ strains. (F) Expression of *PNC1* can extend the lifespan of a strain lacking *MSN2/4*. Average lifespans: wild-type, 23.2 divisions; wild-type + *PNC1,* 28.0; *msn2*Δ/*4*Δ, 22.0; and *msn2*Δ/*4*Δ + *PNC1,* 27.3. Glu, glucose; Rap, rapamycin.

The requirement of *MSN2/4* for CR- and TOR-mediated lifespan extension indicated that CR and TOR might extend lifespan via the same mechanism. We and others have presented evidence that CR works by increasing Sir2p activity [8,14,29], as indicated by increases in telomeric silencing and a reduction in rDNA recombination [7,15,35]. We therefore examined whether rapamycin increased telomeric silencing and reduced rDNA recombination, and whether this was altered by the presence or absence of *MSN2/4* or *PNC1*. We observed that rapamycin increased telomeric silencing in a wild-type strain, but not in strains lacking *MSN2/4* or *PNC1* (Figure S1). Treatment with rapamycin also suppressed rDNA recombination and, again, this effect required *MSN2/4* and *PNC1* (Figure 2A and 2B). The effect of rapamycin on silencing and rDNA recombination was also completely blocked by treating cells with NAM (Figure 2B), indicating that a sirtuin is likely required for this effect. Neither CR nor rapamycin increased protein levels of Sir2p (Figure 2C). Taken together, these data indicate that inhibition of TOR signaling increases the activity of Sir2p and/or another sirtuin.

In addition to Sir2p, S. cerevisiae contains four HST (homolog of sir two) genes, *HST1–4*. We and others have uncovered considerable redundancy in the sirtuin family, with Hst1p and Hst2p able to substitute for Sir2p during CR [15]. Recent studies in our laboratory have demonstrated that overexpression of any one of the sirtuin genes *HST1–4* can suppress rDNA recombination (D. Lamming and M. Latorre-Esteves, unpublished data). Thus, we were not surprised to find that rapamycin could suppress rDNA recombination in a *sir2*Δ *fob1*Δ strain (Figure S2). This result supports a previously published report that inhibition of TOR signaling can extend lifespan in the absence of *SIR2* [17]. Although TOR inhibition can suppress rDNA recombination in the absence of *SIR2,* deletion of both *SIR2* and *HST2* blocked the ability of rapamycin to suppress rDNA recombination (Figure S2). Consistent with these data, we found that rapamycin could extend the lifespan of a *sir2*Δ *fob1*Δ strain but had no effect on the lifespan of a *sir2*Δ *hst2*Δ *fob1*Δ strain (Figure S2). Thus, rather than working through a single sirtuin, we favor a model in which inhibition of TOR signaling suppresses rDNA recombination and promotes longevity by activating multiple sirtuins, including Sir2p and Hst2p.

**Figure 3:**
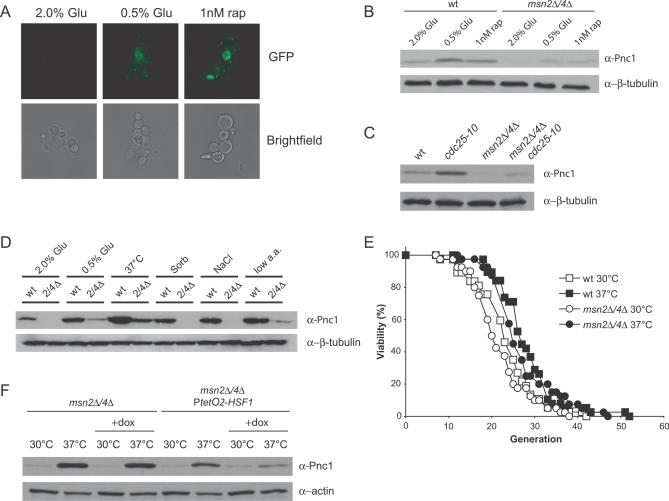
*MSN2/4* Regulate the Expression of *PNC1* (A) Expression of Pnc1p-GFP is induced by CR and by rapamycin. (B) Western blotting shows that CR and rapamycin induce high levels of Pnc1p in an *MSN2/4*-dependent manner. (C) A *cdc25–10* mutant has increased expression of *PNC1* but not if *MSN2/4* are deleted. (D) Increased expression of *PNC1* in response to salt stress, osmotic stress, and amino acid restriction requires *MSN2/4,* but increased expression of *PNC1* in response to heat shock does not*.* (E) *MSN2/4* are not required for lifespan extension by heat shock. Average lifespans: wild-type at 30 °C, 22.2 divisions; wild-type at 37 °C, 26.8; *msn2*Δ/*4*Δ at 30 °C, 20.7; and *msn2*Δ/*4*Δ at 37 °C, 25.8. (F) Heat shock induction of *PNC1* in an *msn2*Δ/*4*Δ strain is dependent upon *HSF1*. *2/4*Δ*, msn2*Δ/*4*Δ; dox, doxycycline; Glu, glucose; low a.a., low amino acids; rap, rapamycin; Sorb, sorbitol; wt, wild-type.

In agreement with this model, *PNC1* was required for lifespan extension by rapamycin (Figure 2D), and overexpression of *PNC1* was sufficient to suppress rDNA recombination and extend lifespan in an *msn2*Δ/*4*Δ background (Figure 2E and 2F). Thus, within the framework of our model, TOR signaling is upstream of *PNC1* and *PNC1* is downstream of *MSN2/4*. The simplest mechanistic explanation is that inhibition of TOR activates *MSN2* and *MSN4,* which then increase the expression of *PNC1*. To test this, we examined the effect of CR and rapamycin on Pnc1p levels in the presence and absence of *MSN2/4*. Both treatments induced Pnc1p-GFP (Figure 3A) as well as native Pnc1p (Figure 3B). In contrast, there were considerably lower levels of Pnc1p in the untreated *msn2*Δ/*4*Δ strain, and Pnc1p levels remained below those of the untreated wild-type strain, even in response to CR or rapamycin (Figure 3B).

Msn2p/4p are normally maintained in the cytoplasm by the activity of PKA. As cAMP levels fall, PKA activity decreases, and dephosphorylated Msn2p/4p relocalize to the nucleus [36]. A *cdc25–10* mutant that has constitutively decreased cAMP/PKA signaling [37] expressed higher levels of Pnc1p than the wild-type strain, and this was *MSN2/4*-dependent (Figure 3C). Upregulation of *PNC1* expression by other stresses was also *MSN2/4*-dependent (Figure 3D), and in agreement with the lifespan data in Figure 1B, single deletion of *MSN2* or *MSN4* did not greatly affect the ability of CR to induce *PNC1* (Figure S3).

We have previously shown that heat shock extends lifespan in a *PNC1*-dependent manner [29], so we were curious whether this was an *MSN2/4*-dependent process. Heat shock induced the expression of *PNC1* and extended lifespan even in the absence of *MSN2/4* (Figure 3D and 3E). To explore the *MSN2/4*-independent mechanism by which heat shock induces *PNC1,* we analyzed the *PNC1* promoter and found putative binding sites for the heat shock factor Hsf1p (positions −251 to −275 and −319 to −353, with respect to the start codon). Since *HSF1* is an essential gene, we used an *msn2*Δ*/4*Δ strain containing doxycycline-repressible *HSF1* to examine the role of Hsf1p in *PNC1* regulation [38]. Repression of *HSF1* largely blocked the ability of heat shock to upregulate *PNC1* (Figure 3F), but it did not affect the ability of CR or rapamycin to induce *PNC1* (data not shown).

**Figure 4:**
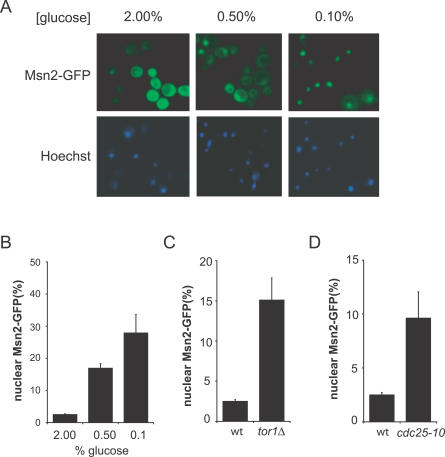
Msn2p Localizes to the Nucleus during CR (A) CR promotes increased nuclear migration of Msn2p-GFP in a dose-dependent manner. (B) Quantification of the percent of cells with predominately nuclear Msn2p-GFP or Msn4p-GFP. (C) Deletion of *TOR1* promotes nuclear localization of Msn2p-GFP. (D) A *cdc25–10* mutant has increased nuclear localization of Msn2p-GFP.

Under standard yeast growth conditions (2% glucose, 30 °C), Msn2p/4p localize predominately to the cytoplasm [36], but in response to a variety of stresses, Msn2p/4p localize to the nucleus, where they activate stress-responsive genes [39]. We observed that CR induced the translocation of Msn2p into the nucleus, and the extent of the translocation was proportional to the degree of CR (Figure 4A and 4B). CR also induced the nuclear localization of Msn4p, but quantification of the nuclear localization indicated that Msn4p was less sensitive to glucose restriction than Msn2p (Figure S4). The reasons for this difference are unknown, but it may allow for differential gene regulation as nutrients are depleted. Nuclear localization of Msn2p also increased in both *tor1*Δ and *cdc25–10* strains (Figure 4C and 4D), in agreement with previous reports [40,41].

**Figure 5:**
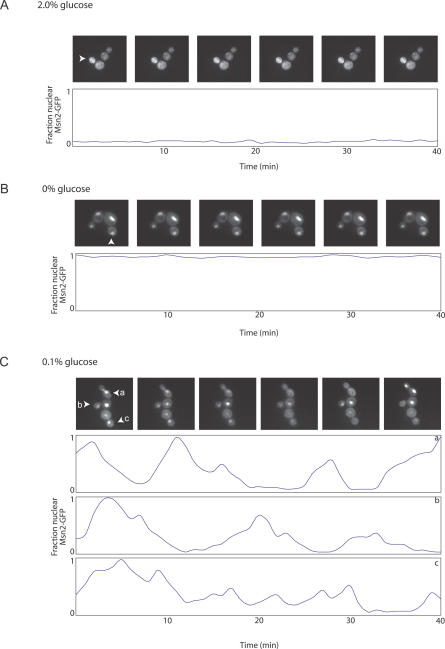
CR Induces Nucleo-Cytoplasmic Oscillations of Msn2p-GFP During CR, Msn2p-GFP oscillates between the cytoplasm and nucleus. (A and B) Cells grown at 2% glucose concentrations (A) exhibit exclusively cytoplasmic localization of Msn2p-GFP. Cells placed in medium containing 0% glucose (B) exhibit exclusively nuclear localization of Msn2p-GFP. For cells in 2% glucose or 0% glucose, a plot of the ratio of average nuclear intensity versus average cytoplasmic intensity for a single representative cell, labeled with an arrowhead in the respective photomicrographs, is shown. (C) During CR conditions, cells exhibit nucleo-cytoplasmic oscillations of Msn2p-GFP with a periodicity of ∼2–3 min. A representative population of cells is shown at 0.1% glucose concentrations. The ratios of average nuclear intensity versus average cytoplasmic intensity for three representative cells, labeled a, b, and c, are shown plotted over the course of 20 min beneath the photomicrographs.

Cells grown in moderate CR conditions (0.5% and 0.1% glucose) showed heterogeneous localization patterns for Msn2p/4p, indicating that Msn2p/4p might be oscillating between the nucleus and cytoplasm, as has recently been noted for cells exposed to light stress or osmotic shock [42]. To determine if this was the case, time-lapse photomicrographs of cells expressing Msn2p-GFP were taken at 30-s intervals during growth under various glucose concentrations. The vast majority of cells grown in 2% glucose showed a cytoplasmic localization of Msn2p (Figure 5A), while cells incubated in medium lacking glucose had exclusively nuclear localization of Msn2p (Figure 5B). These patterns did not change over time. However, in cells grown under intermediate levels of CR (0.1% or 0.5% glucose), nucleo-cytoplasmic oscillations of Msn2p-GFP were observed (Figures 5C and S5). The GFP signal eventually became bleached (data not shown), demonstrating that Msn2p was not simply being degraded in the nucleus and re-synthesized in the cytoplasm but was instead actively being transported in and out of the nucleus [43]. Msn4p-GFP showed a similar oscillatory behavior, and localization of Msn2p or Msn4p under 2% or 0.5% glucose was not altered by a lack of the other transcription factor (data not shown).

**Figure 6:**
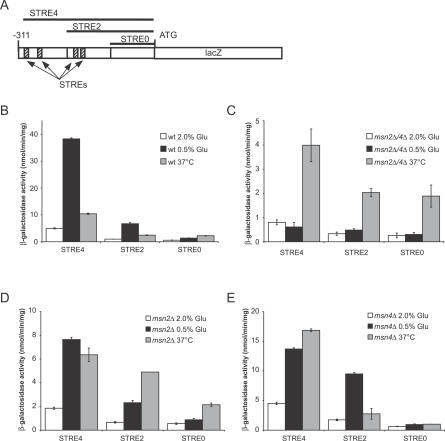
Deletion of *MSN2/4* Abrogates Expression of *LacZ* Reporter Constructs Driven by the *PNC1* Promoter (A) Reporter constructs for analysis of STRE and Msn2p/4p function. (B) β-galactosidase activity of reporter constructs in wild-type (wt) cells grown in 2% YPD (Glu) (white bars), 0.5% YPD (black bars), or 2%YPD at 37 °C (grey bars). (C–E) Activity of reporter constructs in (C) an *msn2*Δ/*4*Δ strain, (D) an *msn2*Δ strain, or (E) an *msn4*Δ strain. Activity is listed as nanomoles of cleaved *o*-nitrophenol-β-D-galactopyranoside substrate per minute per milligram total protein.

The *PNC1* promoter contains four Msn2p/4p-binding sites known as stress response elements (STREs) [32]. To determine whether Msn2p/4p directly regulate *PNC1* in response to CR, we tested several reporter constructs containing different regions of the *PNC1* promoter (Figure 6A). The full-length promoter construct was greatly induced in response to CR (Figure 6B), whereas the reporters lacking one or more of the STREs were induced at significantly lower levels. The construct with no STREs showed no induction. The involvement of *MSN2/4* was confirmed by the finding that CR completely failed to upregulate the reporter in an *msn2*Δ/*4*Δ strain (Figure 6C). The ability of CR to induce the *PNC1* reporter was largely unaffected in single *msn2*Δ or *msn4*Δ mutants (Figure 6D and 6E), which is consistent with the ability of CR to extend the lifespan of single but not double *msn* mutants (see Figure 1A and 1B). Interestingly, deletion of *MSN4* did not affect the expression of the STRE4 reporter construct under 2% glucose, but significantly reduced its response to CR (Figure 6E). In contrast, the STRE2 reporter construct was unaffected by the *MSN4* deletion, indicating that the influence of Msn4p on *PNC1* expression may be mediated by the distal two STRE elements.

We have previously shown that while single deletion of *MSN2* impairs the ability of *PNC1* expression to be induced by stress, single deletion of *MSN4* has little effect (Figure S3). Furthermore, Msn2p localizes to the nucleus more readily in response to decreased glucose concentration (compare Figures 4 and S4), deletion of *MSN2* results in approximately 2-fold less expression of the reporter construct than deletion of *MSN4,* and the STRE2 constructs were less responsive to CR in an *msn2*Δ strain than in an *msn4*Δ strain (Figure 6D and 6E). Together these data support the conclusion that Msn2p is more important than Msn4p for regulating *PNC1* expression in response to CR.

Msn2p also appears to be more important for the response of *PNC1* to heat stress (37 °C). Deletion of *MSN2* resulted in an approximately 2-fold decrease in the expression of the STRE4 reporter construct in response to heat, relative to the wild-type strain (compare Figure 6B and 6D). In contrast, single deletion of *MSN4* actually leads to increased expression of the STRE 4 reporter in response to heat (compare Figure 6B and 6E). This upregulation in the *msn4*Δ strain is likely to be due to *MSN2,* because deletion of *MSN2* in the *msn4*Δ strain resulted in the poorest response of the STRE4 reporter to heat (Figure 6C). The data also indicated that elements present in the STRE4 reporter but not in the STRE2 reporter are responsible for the *MSN2/4*-independent induction of the promoter in response to heat stress. Consistent with this hypothesis, the putative binding sites for Hsf1p identified by our promoter analysis lie within this region.

**Figure 7:**
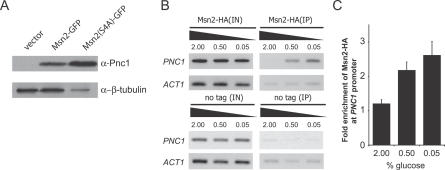
Msn2p Directly Regulates *PNC1* (A) Nuclear localization of Msn2p is sufficient to induce *PNC1.* (B) Chromatin immunoprecipitation analysis of Msn2p-HA binding to the *PNC1* promoter. IN, input; IP, immunoprecipitation. (C) Relative enrichment of Msn2p-HA at the *PNC1* promoter shows increased promoter binding during CR.

Given the relative importance of Msn2p, we next asked if the nuclear localization of Msn2p was sufficient to induce *PNC1*. We expressed a constitutively nuclear mutant of Msn2p, Msn2p(S4A) [39], in an *msn2*Δ and *msn4*Δ mutant, and observed robust induction of Pnc1p (Figure 7A), demonstrating that nuclear localization of Msn2p is sufficient to induce *PNC1* expression. We also noted that this strain grew poorly, in agreement with previous reports that constitutive nuclear localization of Msn2p/4p antagonizes growth [44].

The data thus far strongly supported a model in which CR promotes the binding of Msn2p directly to the *PNC1* promoter, yet there remained the possibility that *PNC1* was regulated by Msn2p indirectly. To distinguish between these two possibilities, we used chromatin immunoprecipitation to determine if Msn2p binds to the *PNC1* promoter in response to CR (Figure 7B). We detected Msn2p at the *PNC1* promoter, and the apparent abundance of Msn2p was proportional to the degree of CR (Figure 7C), which was consistent with the glucose-dependent nuclear localization of Msn2p that we previously observed.

In order to better understand the target specificity of Msn2p/4p in response to CR, we utilized previously published microarray data [45,46] to examine the expression of 82 previously identified STRE-containing genes [47]. We were surprised to find that the genes varied greatly in their responsiveness to conditions of low glucose. Ten genes, including *PNC1,* were highly responsive to small changes in glucose concentration, whereas other STRE-containing genes were responsive only to large changes in glucose concentration or were unresponsive (Table S1). In an effort to understand why some genes are more responsive to Msn2p/4p than others, we analyzed the promoters of these STRE-containing genes. The genes that were less responsive to low glucose averaged fewer STRE elements than the highly responsive genes and, on average, had binding sites approximately 50–60 base pairs further from the start of the coding sequence than genes that responded to low glucose (Table S2). We speculate that the placement of transcription factor binding sites at varying distances from the promoter may be a conserved mechanism for the differential regulation of stress-induced genes.

Because our work indicated that two major longevity pathways, namely CR and TOR, promote lifespan by inducing the expression of *PNC1,* we wondered whether other yeast longevity genes also modulate *PNC1* expression. Deletion of the glycolysis pathway gene *HXK2* extends lifespan and has been proposed as a genetic mimic of CR [15,17,37]. Consistent with this, an *hxk2*Δ strain had higher levels of *PNC1* (Figure S6), placing it upstream of *PNC1.* Similarly, there are abundant data linking the Snf1p/AMPK pathway to yeast longevity. The yeast homolog of AMPK, Snf1p, phosphorylates Msn2p in response to glucose deprivation, is regulated by TOR, and influences lifespan by modulating ERC formation [48,49]. Deletion of *SNF4,* an activator of Snf1p, has been shown to extend lifespan, whereas deletion of *SIP2,* a repressor of Snf1p, shortens lifespan [49]. Surprisingly, we saw no evidence for involvement of the SNF pathway *(SNF1, SNF4,* or *SIP2)* in the regulation of *PNC1* (Figure S6), indicating that the activity of Snf1p/AMPK regulates ERC formation independently of *PNC1*. Furthermore, CR induced *PNC1* expression equally well in wild-type and *snf1*Δ mutant strains (Figure S6).

We also examined the potential role of *ADR1,* a transcription factor that is regulated by *SNF1*/AMPK and that we suspected from our promoter analysis of *PNC1* might regulate *PNC1* [50]. We found that *ADR1* was not required for the induction of *PNC1* by CR (Figure S6 and data not shown), and that overexpression of *ADR1,* or expression of a constitutively active form of Adr1p, did not induce expression of *PNC1* (Figure S6)*.* Together, these data show that while attenuation of TOR signaling, PKA activity *(cdc25–10),* or glucose metabolism (*hxk2*Δ) extends replicative lifespan, ostensibly by mimicking the effects of CR, the SNF pathway regulates lifespan via a *PNC1*-independent mechanism.

## Discussion

**Figure 8:**
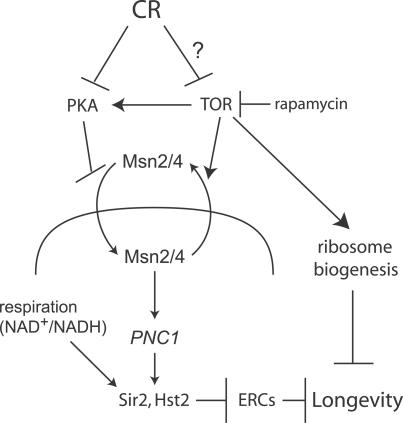
Model of How TOR Signaling and CR Regulate Yeast Replicative Lifespan CR and TOR signaling regulate the nuclear localization of Msn2p/4p. When localized to the nucleus, Msn2p/4p promote the transcription of *PNC1,* a nicotinamidase. The removal of NAM by Pnc1p increases the activity of sirtuins, including Sir2p and Hst2p, which promote longevity by stabilizing the yeast rDNA array and preventing the formation of ERCs. TOR signaling may also regulate lifespan by sirtuin-independent pathways, such as the regulation of ribosomal biogenesis.

How CR delays aging and extends the lifespan of various species is poorly understood. In this study, we have connected two sections of the yeast CR pathway, namely the cytoplasmic components (the glucokinase/cAMP/PKA pathway) and the nuclear components (Pnc1p*,* Sir2p, and ERCs). Furthermore, we have shown that TOR signaling, which was previously thought to regulate lifespan independently of sirtuins and ERCs, actually governs the activity of the sirtuins and suppresses rDNA recombination (Figure 8). This provides additional support to the theory that CR extends replicative lifespan, at least in part, by activating sirtuins.

We also demonstrate that the induction of *PNC1* in response to numerous stresses is largely controlled by the transcription factors Msn2p and Msn4p. Under conditions of high salt or sorbitol, *PNC1* expression is increased in a manner that is completely dependent on *MSN2/4* (Figure 3C). While we have linked the increase in Pnc1p levels during heat stress in an *msn2*Δ/*4*Δ strain to the transcription factor Hsf1p (Figure 3F), we did not observe a role for Hsf1p in the response to CR or low amino acids.

There must be additional factors that control the expression of *PNC1,* because an increase in Pnc1p levels still occurs in an *msn2*Δ/*4*Δ strain grown in 0.5% glucose or in medium with low amino acids. One possibility is that *PNC1* is co-activated by Gcr1p, a transcriptional activator with potential binding sites ∼500 base pairs upstream of the *PNC1* start codon*. GCR1* regulates glycolytic enzyme genes, ribosomal gene synthesis, and trehalose/glycogen metabolism [51,52], making it an interesting candidate for future analysis, although we note that any such analysis is complicated by the severe growth defect of a *gcr1*Δ strain.

In contrast to our study, a previous study utilizing a *cdc25–10* mutant as a mimic of CR found that replicative lifespan extension of a PSY316 strain can occur in the absence of *MSN2/4* [14]. A recent study has shown that PSY316 may differ substantially from other yeast strains in terms of Sir2p-mediated lifespan extension [18], and our data may reflect that difference. We favor the notion that while the *cdc25–10* mutation mirrors aspects of CR, such as lower PKA activity and increased lifespan, it does not fully replicate it.

A previous study has shown that inhibition of TOR signaling can extend lifespan, even in the absence of *SIR2* [17]. In agreement with this data, we find that treatment with rapamycin can suppress rDNA recombination and extend lifespan in a *sir2*Δ *fob1*Δ strain (Figure S2). Yeast contain four additional sirtuin genes *(HST1–4),* some of which can compensate for the lack of Sir2p during CR [15]. Under the conditions and with the strain used in this study, we have observed that rapamycin no longer lowers rDNA recombination or promotes longevity if both *SIR2* and *HST2* are deleted (Figure S2), implying that these two genes are primarily responsible for the effect. However, a W303 *sir2*Δ *hst2*Δ *fob1*Δ strain has a high rate of rDNA recombination and a short lifespan, which may serve to obscure the role of additional sirtuins or other mediators in the response to TOR inhibition. In fact, overexpression of *PNC1* in a wild-type strain lowers rDNA recombination more than in a strain lacking *MSN2/4*, which may indicate that genes downstream of *MSN2/4* besides *PNC1* also function to repress rDNA recombination. These alternative pathways may be especially important when glucose concentrations are extremely low [53] and may include pathways that directly regulate rDNA stability, such as *RPD3*-dependent loading of condensin onto the rDNA array in response to nutrient signaling [54]. TOR signaling also promotes the synthesis of ribosomal proteins, and downregulation of ribosomal biogenesis can extend the lifespan of both yeast [17] and C. elegans [22,23]. These data suggest that TOR signaling may act to promote lifespan via multiple pathways that act in parallel to promote longevity (Figure 8).

Our analysis of the responsiveness of STRE-containing genes found ten genes, including *PNC1,* that are upregulated more than 2-fold in response to a slight decrease in the glucose concentration (2% to 1.75%) (Table S1). In general, the genes in this category are highly sensitive to environmental stresses, including heat shock and osmotic stress [46]. We speculate that other genes in this category, which includes both metabolic and heat shock genes, may also play a role in lifespan extension. Heat shock proteins in particular have been shown to promote longevity in numerous organisms, and are upregulated during CR in rodents [55,56].

Interestingly, *MSN2/4* have also been shown to be required for the extension of yeast chronological lifespan [57]. *MSN2/4* are responsible for the activation of numerous stress-responsive genes, including the superoxide dismutase *SOD2,* a gene that promotes chronological lifespan [34]. Yet, overexpression of *SOD2* shortens replicative lifespan, and it has been demonstrated that deletion of *MSN2/4* can actually lead to increases in replicative lifespan [58]. Even though we saw no such effect (Figure 1A), perhaps because of a difference in strain background, there may be a reciprocal relationship between replicative and chronological lifespan. A recent study showed that deletion of *SIR2* can extend chronological lifespan in several strains [59], and we have observed that overexpression of *SIR2* or *HST2* shortens chronological lifespan in W303 (unpublished data).

The identification of the stress response factors Msn2p/4p as key components of the CR pathway in yeast supports two theories about CR. The first is known as the hormesis hypothesis of CR, which states that CR is a mild biological stress that provides health benefits because it activates an organism's defenses against adversity [60,61]. The second hypothesis is that the promoter elements of key longevity genes are just as important as the longevity genes themselves [28,62]. These promoters serve as sensors of the organism's environment by accepting different and additive inputs from environmentally sensitive transcription factors. The existence of short DNA sequences that dictate longevity could explain how new lifespans evolve so rapidly in response to a new ecological niche. Theoretically, if a transcription factor binding site regulates a key longevity gene, then a single base change might be sufficient to alter how long a species lives in response to an environmental condition.

In contrast to previous suggestions, we find that TOR and sirtuin signaling are components of the same longevity pathway that extends yeast replicative lifespan by stabilizing the repetitive rDNA (Figure 8). Given the high degree of functional conservation of TOR and sirtuins between yeast and higher organisms, and the recent discovery of a role for mammalian sirtuins in DNA repair [63], the findings in this study raise the possibility that the mammalian TOR pathway influences sirtuin activity and that together they may promote the health and longevity of mammals.

## Materials and Methods

### Yeast strains and plasmids.

W303AR *MAT* **a**, W303AR *MAT* **a** *pnc1::kan^r^,* W303AR *PNC1-GFP::kan^r^*, and W303AR *SIR2-3xHA::kan^r^* have been previously described [24,29,64]. Gene disruptions in W303AR *MAT* **a** were achieved by replacing the wild-type genes with the *kan^r^*, *hph^r^*, or *nat^r^* marker as described [65,66] and verified by PCR using oligonucleotides flanking the genes. *PNC1* was overexpressed as previously described [29]. W303AR *cdc25–10* was created by replacing the endogenous copy of *CDC25* with a plasmid-borne copy of *cdc25–10* (the kind gift of S. J. Lin) as previously described [37]. pPNC1-STRE.4, pPNC1-STRE.2, and pPNC1-STRE.0 constructs and the pAdh-Msn2p-GFP/HA constructs were kindly provided by M. Ghislain [33] and C. Schuller [36], respectively. Msn4p-GFP and Msn2p(S4A)-GFP constructs were the kind gift of M. Jacquet. Plasmids for expression of Adr1p were obtained from E. Young. BY4741 deletions in this background were from F. Winston and P. Silver (Harvard), BY4742 and BY4742 *hxk2::kan^r^* were from B. Kennedy [18], W303 *msn2*Δ/*4*Δ and W303 *msn2*Δ/*4*Δ *tetO-HSF1* were from H. Nelson [38]. All primer sequences, strains, and plasmid maps are available upon request.

### Medium.

Yeast were grown in yeast peptone dextrose (YPD) medium supplemented with an additional 0.015% w/v adenine, histidine, leucine, tryptophan, and uridine, and containing 2% w/v glucose during normal growth and 0.5% glucose for CR unless otherwise stated. For growth in low amino acid medium, synthetic complete medium containing 0.03 % w/v essential amino acids and 2% glucose was used. Strains were pre-grown overnight at 30 °C. The following day, cells were inoculated at an O.D. 600 = 0.1 and grown until log phase of growth was attained during the various conditions mentioned (O.D. 600 = 0.7). For treatment with rapamycin or heat shock, cells were grown for 2 h untreated, at which point rapamycin was added to a final concentration of 1 nM, or cells were moved to 37 °C, and cells were then grown for an additional 2 h.

### Recombination and lifespan assays.

rDNA recombination rates were determined by determining the frequency of loss of *ADE2* in the rDNA of strain W303AR as previously described [15,29]. For rapamycin recombination assays, cells were grown for 2 h without rapamycin, followed by growth with 1 nM rapamycin for 2 h. More than 6,000 colonies were examined for each strain. Results are average values and standard deviation of at least three experiments. For replicative lifespan analyses, strains were pre-grown overnight on YPD plates unless otherwise noted. All lifespan analyses were carried out by using micromanipulation as previously described [14], and all micromanipulation dissections, including for cells grown under heat stress (37 °C), were carried out at laboratory temperature. For cells treated with rapamycin, yeast that growth-arrested in the G_1_ phase of the cell cycle due to toxicity [67] within the first nine divisions were excluded from the datasets. Statistical analysis was carried out using the JMP-IN statistics package (SAS, http://www.sas.com/). Wilcoxon rank-sum test *p*-values were calculated for each pair of lifespans, as shown in Table S3.

### Fluorescence microscopy and imaging.

For the observation of nuclear migration of Msn2p-GFP or Msn4p-GFP, yeast were grown in YPD for 30 min at 1%, 0.5%, 0.1%, and 0.05% glucose (w/v), after first pre-growing to log phase in 2% glucose. Nuclei in live cells were stained with Hoechst #33342 (Sigma-Aldrich, http://www.sigmaaldrich.com/). Time-lapse photomicrographs were captured every 30 s using 1-s exposures. Image analysis was performed using the imageJ software package (National Institutes of Health, http://www.nih.gov/) in order to calculate the ratios of average nuclear intensity versus average cytoplasmic intensity.

### β-galactosidase reporter assays and protein extraction.

Whole cell extracts were used to assay β-galactosidase activity as described [33]. Enzymatic activity is expressed as nanomoles *o*-nitrophenol-β-D-galactopyranoside cleaved per minute per milligram total protein.

### Western analysis and antibodies.

Rabbit anti-Pnc1p polyclonal antibodies were generated by immunization of rabbits (Covance, http://store.crpinc.com/) with recombinant protein, and fresh serum was used at a dilution of 1:5,000. Mouse monoclonal anti-β-tubulin antibody (MAB3408, clone KMX-1, Upstate, http://www.upstate.com/), mouse monoclonal anti-actin antibody (Upstate/Chemicon MAB1501) and polyclonal rabbit anti-HA antibody (Abcam, http://www.abcam.com/) were used at a dilution of 1:1,000. Anti-rabbit (Amersham, http://www.amersham.com/) and anti-mouse (Amersham) horse radish peroxidase–conjugated antibodies were used at dilutions of 1:7,000.

### Chromatin immunoprecipitation assay and PCR analysis.

The chromatin immunoprecipitation procedure was a modification of the method described by Strahl-Bolsinger et al. [68]. Changes to the protocol are as follows: 100 ml of cells (2.0 × 10^7^ cells/ml) was cross-linked with 2% formaldehyde for 15 min at room temperature. Glycine was added to a final concentration of 250 mM, and the incubation continued for an additional 5 min. The suspension was sonicated seven times for 10 s with the amplitude set at 30% using a Branson model 450 digital sonifier (Branson, http://www.bransonultrasonics.com/). The suspension was clarified by centrifugation for 5 min, maximum setting, at 4 °C in a microcentrifuge. Samples were incubated on ice for 2 min between pulses. Then 1 μl of RNase (10 μg/ul) was added to samples, and they incubated for 30 min at 37 °C. Afterwards, sheared chromatin was purified using QIAquick spin columns (Qiagen, http://www1.qiagen.com/). Then 250 μl of supernatant was incubated with 15 μl of rabbit anti-HA antibody (Abcam). For the PCR analysis, the actin control primers used were as follows: ACT1-Chip-Fwd, GCCTTCTACGTTTCCATCCA [69], and ACT1-Chip-Rev, GGCCAAATCGATTCTC AAAA [69]. The *PNC1* promoter primers used were as follows: Pnc1p-Chip-Fwd, GATCAAGGTGGCACACAGGG, and Pnc1p-Chip-Rev, ATACATAGTGGGCCAAACGG. The PCR protocol used was one cycle with 2 min at 95 °C, 30 s annealing at 55 °C, and a 1-min extension at 72 °C, followed by 30 cycles with 30 s at 95 °C, 30 s annealing at 55 °C, and 1-min extension at 72 °C. A final extension was performed for 4 min at 72 °C. Specific binding of Msn2p-HA to the endogenous *PNC1* promoter *(PNC1p)* was analyzed by calculating the ratio of the percent IP of *PNC1p* to the percent IP of *ACT1,* using the V4.2.2 Quantity One 1-D analysis package (Bio-Rad, http://www.bio-rad.com/).

## Supporting Information

Figure S1 Inhibition of TOR Signaling by 1 nM Rapamycin Increases Telomeric Silencing (433 KB PDF)

Figure S2 Suppression of rDNA Recombination by Rapamycin Does Not Depend upon a Single Sirtuin (601 KB PDF)

Figure S3 Effect of Single Deletions of *MSN2* or *MSN4* on *PNC1* Expression during Stresses (7.3 MB PDF)

Figure S4 Msn4p Localizes to the Nucleus during CR (1.2 MB PDF)

Figure S5 CR Induces Nucleo-Cytoplasmic Oscillations of Msn2p-GFP (509 KB PDF)

Figure S6 Snf1p Signaling Does Not Regulate *PNC1*
(509 KB PDF)

Table S1 Expression of STRE-Containing Genes during the Diauxic Shift (757 KB PDF)

Table S2 STRE Promoter Analysis (12 KB PDF)

Table S3 Lifespan Analysis Wilcoxon rank-sum test *p*-values were calculated for each pair of lifespans using JMP-IN statistical analysis software.
(23 KB PDF)

## Abbreviations

CR: calorie restriction
ERC: extrachromosomal ribosomal DNA circle
NAM: nicotinamide
rDNA: ribosomal DNA
STRE: stress response element
YPD: yeast peptone dextrose
