# Supplementary material for: MSN2 and MSN4 Link Calorie Restriction and TOR to Sirtuin-Mediated Lifespan Extension in Saccharomyces cerevisiae
Source: PLoS Biol. 2007 Oct 2;5(10):e261. doi: 10.1371/journal.pbio.0050261 (PMC1994990; doi:10.1371/journal.pbio.0050261)
Supplement: Supplementary file 1 [file pbio.0050261.sg001.pdf]

Figure S1

A

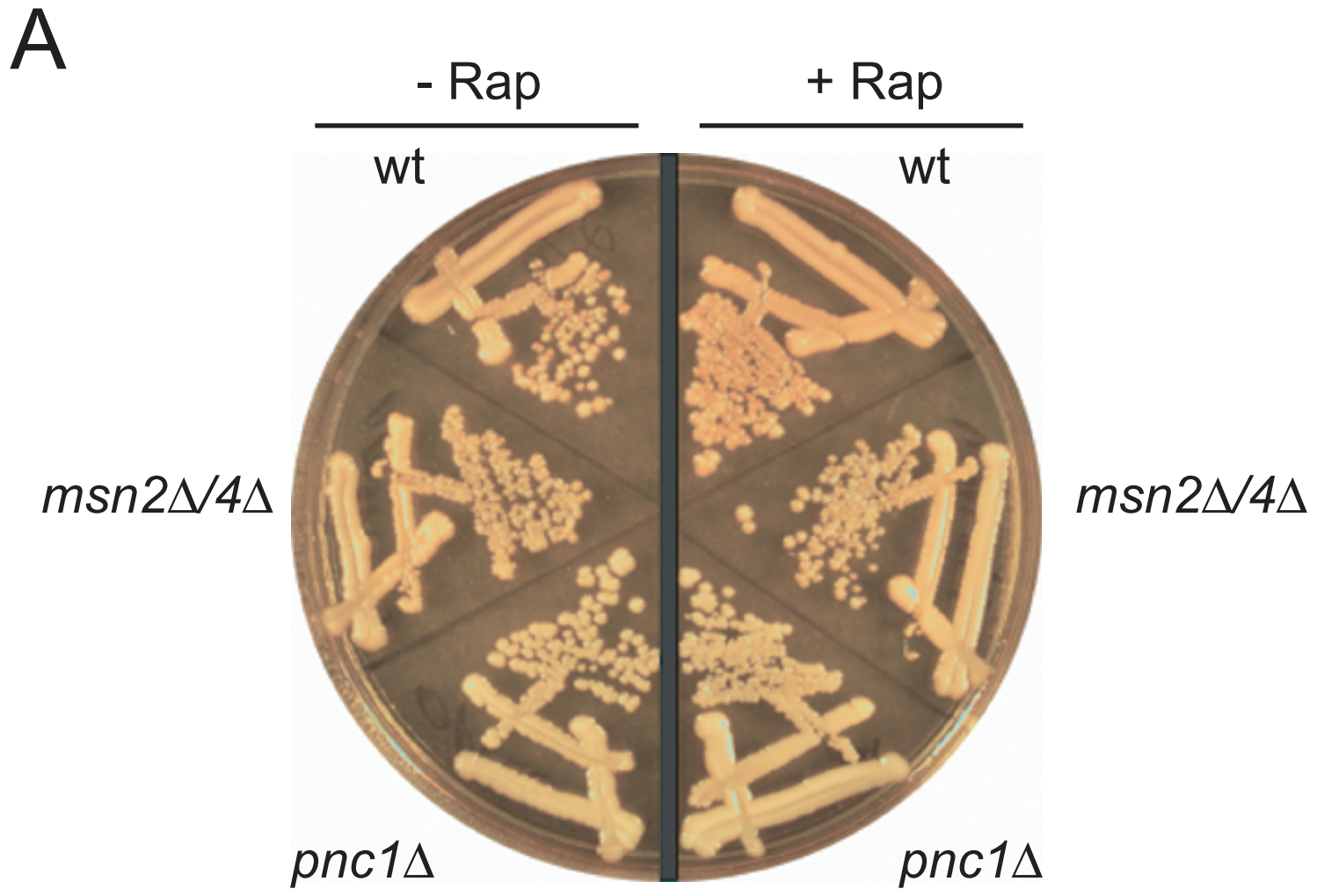

- Rap
+ Rap
wt
wt
msn2Δ/4Δ
msn2Δ/4Δ
pnc1Δ
pnc1Δ

B

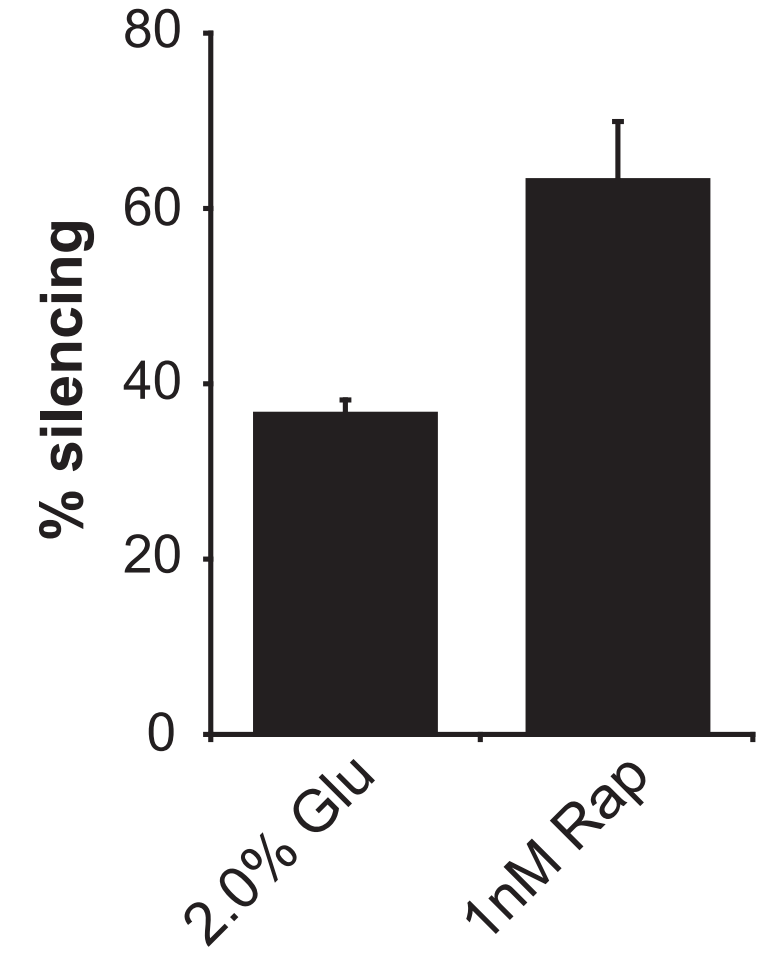

% silencing
80
60
40
20
0
2.0% Glu
1nM Rap
