# Supplementary material for: MSN2 and MSN4 Link Calorie Restriction and TOR to Sirtuin-Mediated Lifespan Extension in Saccharomyces cerevisiae
Source: PLoS Biol. 2007 Oct 2;5(10):e261. doi: 10.1371/journal.pbio.0050261 (PMC1994990; doi:10.1371/journal.pbio.0050261)
Supplement: Supplementary file 2 [file pbio.0050261.sg002.pdf]

Figure S2

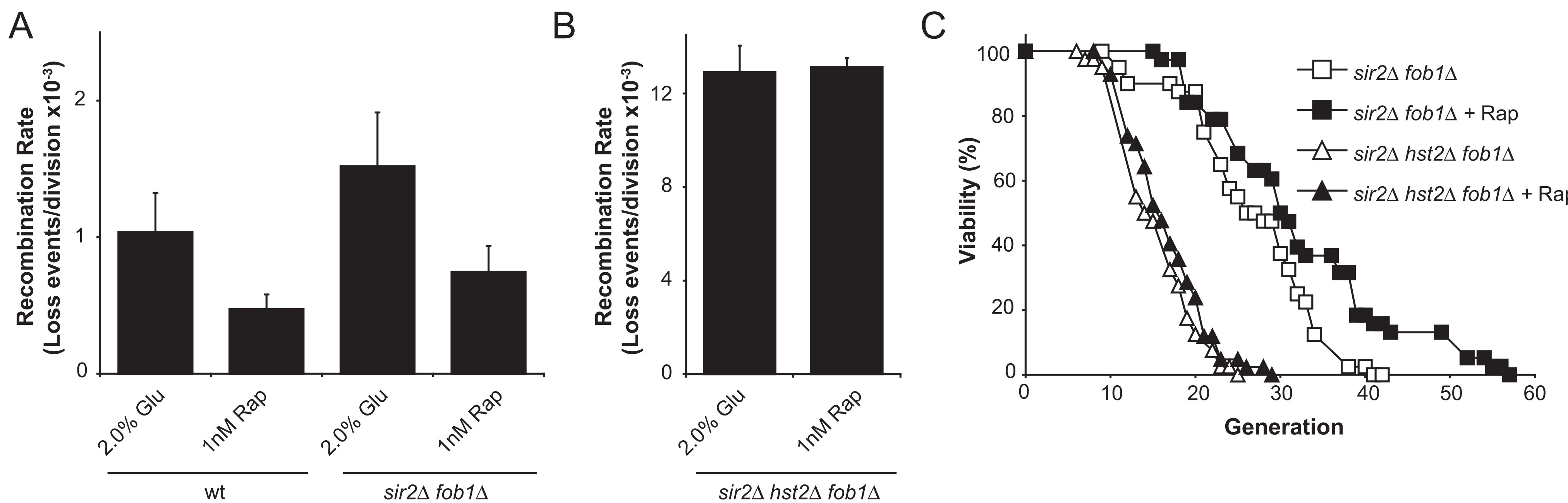

A
Recombination Rate
(Loss events/division x10^{-3})
0
1
2
2.0% Glu
1nM Rap
2.0% Glu
1nM Rap
wt
sir2Δ fob1Δ
B
Recombination Rate
(Loss events/division x10^{-3})
0
4
8
12
2.0% Glu
1nM Rap
sir2Δ hst2Δ fob1Δ
C
Viability (%)
0
20
40
60
80
100
0
10
20
30
40
50
60
Generation
sir2Δ fob1Δ
sir2Δ fob1Δ + Rap
sir2Δ hst2Δ fob1Δ
sir2Δ hst2Δ fob1Δ + Rap
