# Supplementary material for: MSN2 and MSN4 Link Calorie Restriction and TOR to Sirtuin-Mediated Lifespan Extension in Saccharomyces cerevisiae
Source: PLoS Biol. 2007 Oct 2;5(10):e261. doi: 10.1371/journal.pbio.0050261 (PMC1994990; doi:10.1371/journal.pbio.0050261)
Supplement: Supplementary file 3 [file pbio.0050261.sg003.pdf]

Figure S3

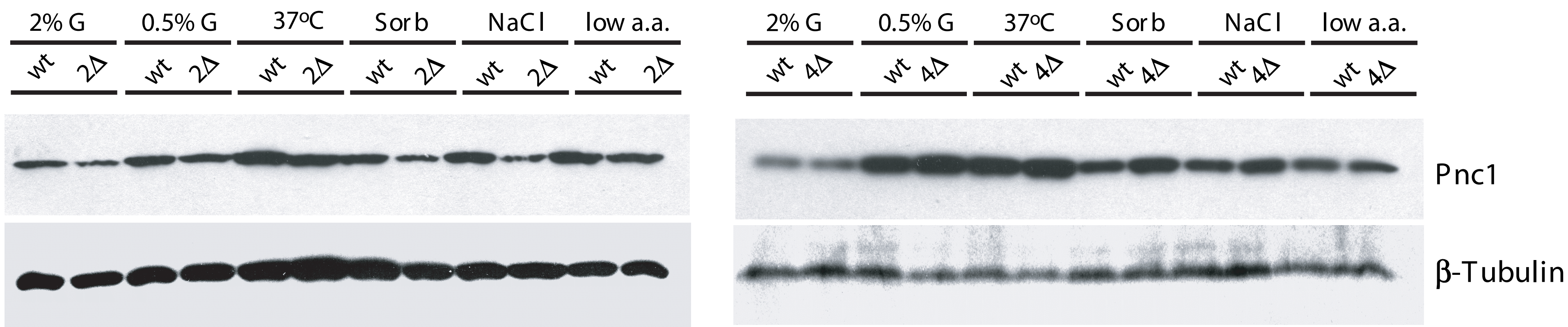

2% G
0.5% G
37ºC
Sor b
NaC l
low a.a.
wt
2Δ
2% G
0.5% G
37ºC
Sor b
NaC l
low a.a.
wt
4Δ
Pnc1
β-Tubulin
