# Supplementary material for: MSN2 and MSN4 Link Calorie Restriction and TOR to Sirtuin-Mediated Lifespan Extension in Saccharomyces cerevisiae
Source: PLoS Biol. 2007 Oct 2;5(10):e261. doi: 10.1371/journal.pbio.0050261 (PMC1994990; doi:10.1371/journal.pbio.0050261)
Supplement: Supplementary file 4 [file pbio.0050261.sg004.pdf]

Figure S4

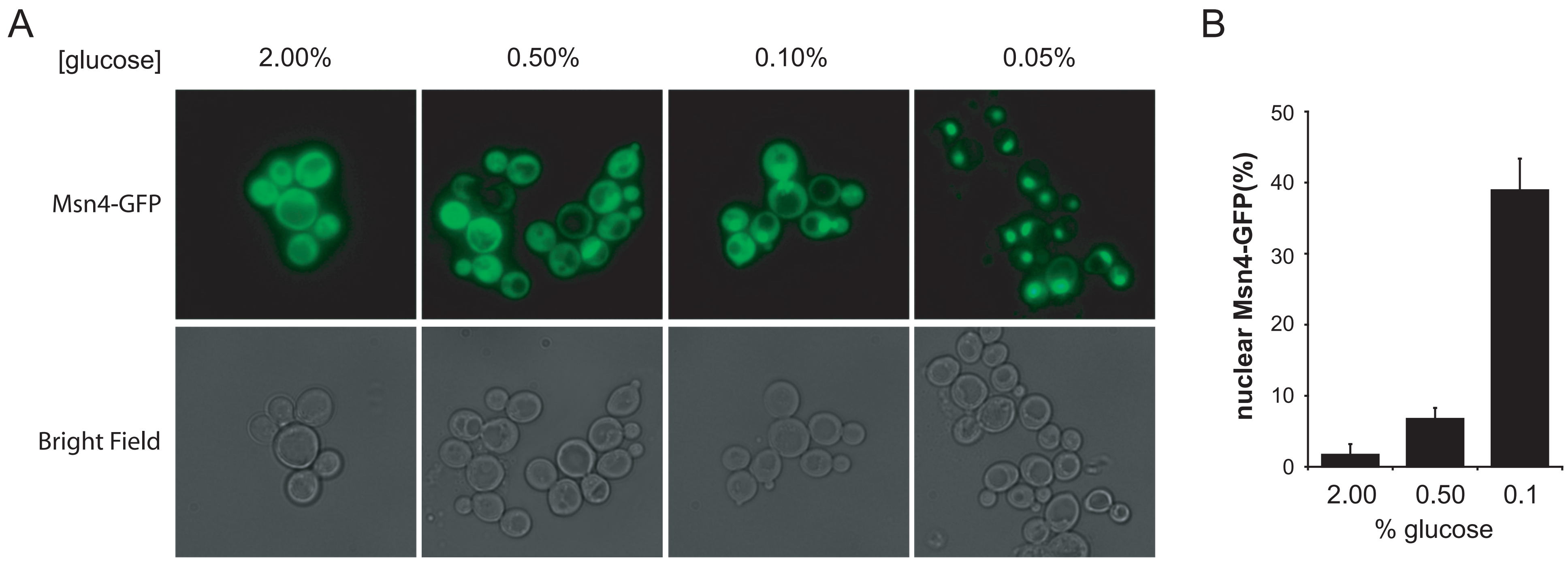
A
[glucose]
2.00%
0.50%
0.10%
0.05%
Msn4-GFP
Bright Field
B
nuclear Msn4-GFP(%)
50
40
30
20
10
0
2.00
0.50
0.1
% glucose
