# Supplementary material for: MSN2 and MSN4 Link Calorie Restriction and TOR to Sirtuin-Mediated Lifespan Extension in Saccharomyces cerevisiae
Source: PLoS Biol. 2007 Oct 2;5(10):e261. doi: 10.1371/journal.pbio.0050261 (PMC1994990; doi:10.1371/journal.pbio.0050261)
Supplement: Supplementary file 5 [file pbio.0050261.sg005.pdf]

Figure S5

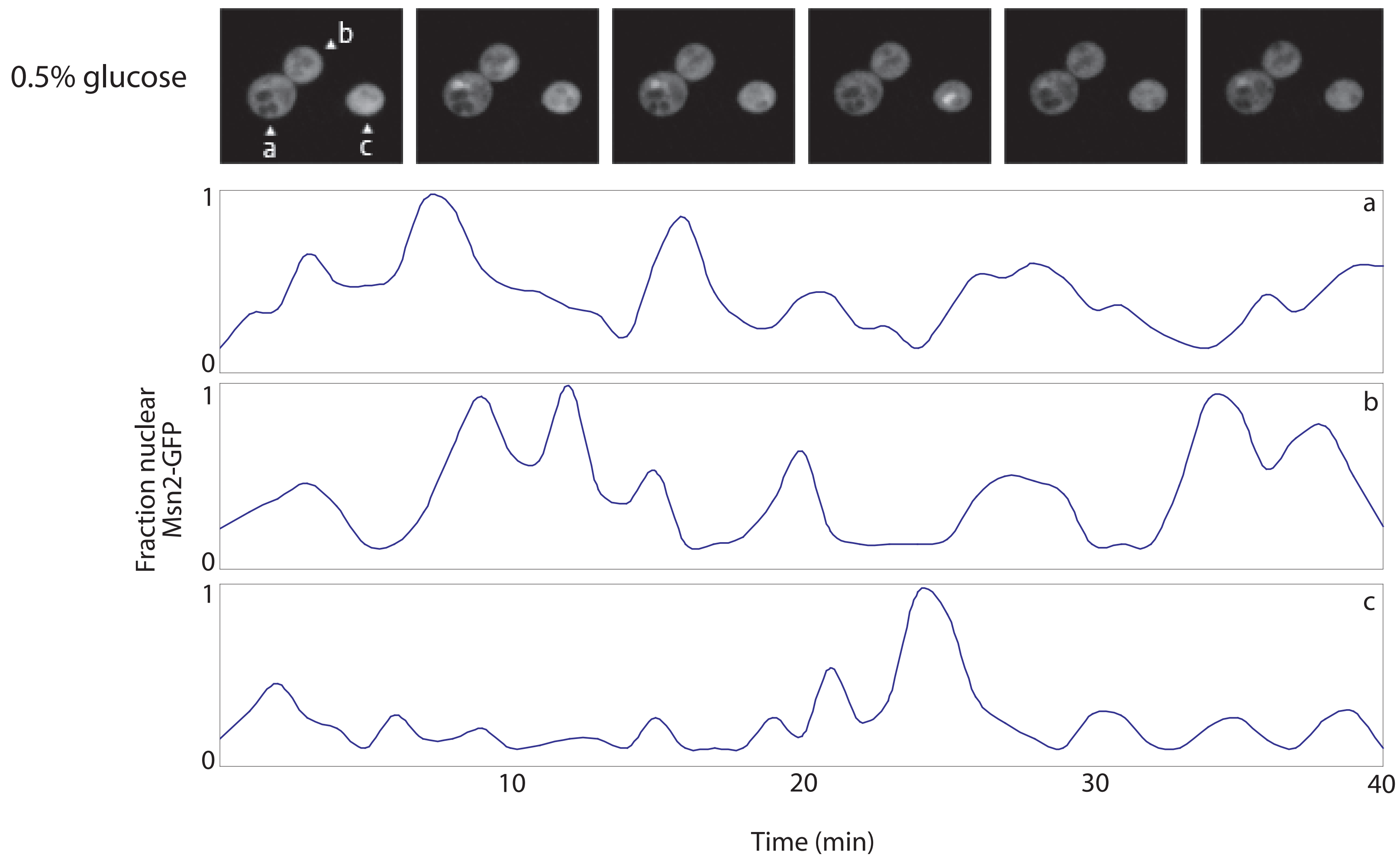

0.5% glucose
b
a
c
1
0
a
1
0
b
1
0
c
Fraction nuclear Msn2-GFP
10
20
30
40
Time (min)
