# Supplementary material for: MSN2 and MSN4 Link Calorie Restriction and TOR to Sirtuin-Mediated Lifespan Extension in Saccharomyces cerevisiae
Source: PLoS Biol. 2007 Oct 2;5(10):e261. doi: 10.1371/journal.pbio.0050261 (PMC1994990; doi:10.1371/journal.pbio.0050261)
Supplement: Supplementary file 6 [file pbio.0050261.sg006.pdf]

Figure S6

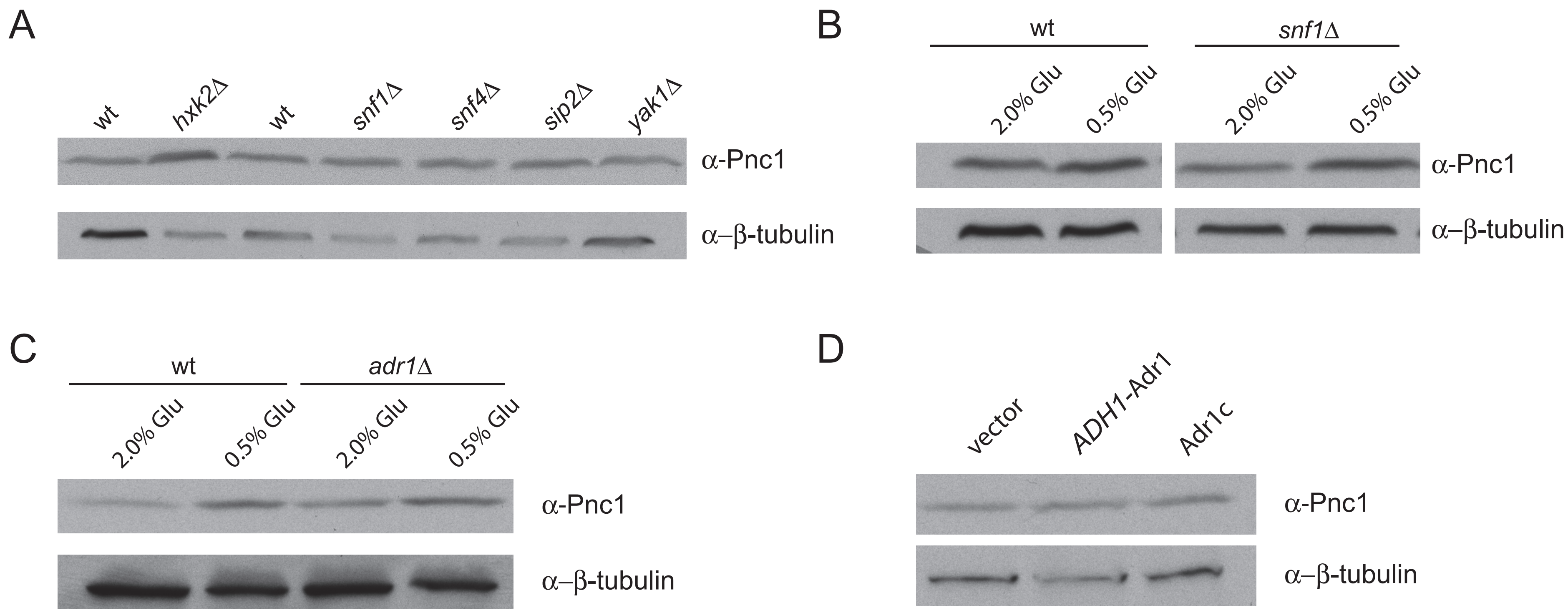
A
wt
hxk2Δ
wt
snf1Δ
snf4Δ
sip2Δ
yak1Δ
α-Pnc1
α–β-tubulin
B
wt
snf1Δ
2.0% Glu
0.5% Glu
2.0% Glu
0.5% Glu
α-Pnc1
α–β-tubulin
C
wt
adr1Δ
2.0% Glu
0.5% Glu
2.0% Glu
0.5% Glu
α-Pnc1
α–β-tubulin
D
vector
ADH1-Adr1
Adr1c
α-Pnc1
α–β-tubulin
