# Supplementary material for: MSN2 and MSN4 Link Calorie Restriction and TOR to Sirtuin-Mediated Lifespan Extension in Saccharomyces cerevisiae
Source: PLoS Biol. 2007 Oct 2;5(10):e261. doi: 10.1371/journal.pbio.0050261 (PMC1994990; doi:10.1371/journal.pbio.0050261)
Supplement: Supplementary file 7 [file pbio.0050261.st001.pdf]

Supplemental Table 1

A

	classification	Fold Induction at 13.5 hrs (1.75% glucose)	Fold Induction at 18.5 hrs (0.1% glucose)	Number of STREs	Nearest STRE position
GSY2	metabolism	3.73	10.41	2	-326
PGM2	metabolism	3.34	9.13	3	-255
HSP12	cell rescue	2.99	12.13	5	-184
PRC1	protein destination	2.35	3.27	2	-160
PNC1	unclassified	2.23	7.06	4	-287
COX5b	metabolism	2.14	7.94	2	-163
TPK1	signal transduction	2.08	3.29	3	-129
TSL1	metabolism	2.06	3.18	4	-160
TPS2	metabolism	2.04	4.06	5	-303
HSP26	cell rescue	2.01	12.38	4	-320

B

	classification	Fold Induction at 13.5 hrs (1.75% glucose)	Fold Induction at 18.5 hrs (0.1% glucose)	Number of STREs	Nearest STRE position
YNL194c	unclassified	0.89	14.93	4	-284
HSP42	protein destination	1.80	12.38	3	-180
CTT1	cell rescue	1.66	12.30	2	-358
YKL026c	metabolism	1.20	12.04	2	-242
YGR043c	metabolism	0.93	11.24	3	-166
ACH1	metabolism	1.08	6.59	4	-130
HXK1	metabolism	1.99	5.78	5	-340
HSP104	cell rescue	1.79	5.28	3	-166
HSP78	protein destination	1.71	5.17	3	-136
GAC1	metabolism	1.21	5.10	1	-459
GLK1	metabolism	1.72	4.92	3	-110
YHR195w	unclassified	1.10	4.26	2	-188
NTH1	metabolism	1.82	4.14	3	-150
YDL085w	metabolism	0.92	4.14	2	-133
YIR038c	GTT1	1.58	3.89	3	-110
YAL061w	metabolism	0.81	3.73	2	-126
CYC7	cellular organization	1.03	3.73	3	-193
YPL004c	unclassified	1.44	3.61	3	-178
YBR149w	metabolism	1.30	3.58	2	-82
TPS1	metabolism	1.47	3.41	6	-233
YAK1	signal transduction	0.87	3.14	3	-208
YKR076w	unclassified	1.18	3.12	2	-112
YLR168c	protein destination	1.12	2.95	2	-244
XBP1	transcription	1.21	2.95	3	-322
YNR014w	unclassified	0.69	2.83	4	-220
ACS1	metabolism	0.88	2.77	2	-240
YPL230w	transcription	1.34	2.68	3	-323
YIL124w	metabolism	1.88	2.62	2	-90
YKL094w	unclassified	1.13	2.53	2	-127
YMR322c	unclassified	0.79	2.50	2	-145
YLR312c	unclassified	1.13	2.39	2	-264

YIL113w	signal transduction	1.03	2.36	2	-126
POR2	cellular organization	1.05	2.23	2	-385
RAS2	signal transduction	1.05	2.23	5	-330
YIR016w	unclassified	0.98	2.17	2	-153
YNL077w	protein destination	0.80	2.17	2	-384
YMR031c	unclassified	1.31	2.14	2	-180
MDH2	metabolism	0.74	2.11	3	-184

C

	classification	Fold Induction at 13.5 hrs (1.75% glucose)	Fold Induction at 18.5 hrs (0.1% glucose)	Number of STREs	Nearest STRE position
SSA4	protein destination	0.89	1.97	3	-354
YDR479c	unclassified	0.95	1.85	2	-128
YDL025c	signal transduction	1.22	1.77	3	-267
YKR075c	unclassified	0.81	1.66	2	-425
YKL107w	unclassified	0.75	1.62	2	-136
GLC7	metabolism	1.13	1.55	2	-250
PIG2	metabolism	0.84	1.55	2	-325
YGR086c	unclassified	0.95	1.49	3	-200
YLR390w	unclassified	1.02	1.47	2	-100
YPL003w	protein destination	0.90	1.37	3	-165
MDJ1	protein destination	0.90	1.37	4	-350
RPB4	transcription	1.06	1.36	3	-357
YLL020c	unclassified	0.81	1.35	3	-350
YGL036w	unclassified	0.82	1.35	4	-349
SGA1	metabolism	0.94	1.34	4	-180
YLR108c	unclassified	0.57	1.33	3	-200
SPS100	cellular organization	0.81	1.32	2	-145
TPS3	metabolism	1.19	1.29	2	-211
CPH1	protein destination	1.21	1.27	2	-379
DAL1	metabolism	0.66	1.24	2	-94
YMR106c	cell rescue/yku80	0.68	1.16	2	-440
YML101c	unclassified	0.73	1.06	4	-210
DIG2	cellular organization	1.07	1.05	2	-382
YBR004c	protein destination	1.09	1.02	2	-465
YLL025w	unclassified	1.01	1.01	2	-477
SSA1	protein destination	0.99	1.01	2	-155
PAU6	cell rescue	1.01	0.97	2	-330
PTP2	signal transduction	0.69	0.94	2	-110
GPH1	metabolism	1.08	0.87	3	-327
YLR177w	unclassified	0.90	0.85	4	-117
RET1	transcription	0.64	0.79	2	-353
YEL045c	unclassified	0.63	0.76	2	-443
STE23	protein destination	0.75	0.72	3	-268
YNR013c	metabolism/Pho91	0.75	0.60	4	-456
