# Supplementary material for: MSN2 and MSN4 Link Calorie Restriction and TOR to Sirtuin-Mediated Lifespan Extension in Saccharomyces cerevisiae
Source: PLoS Biol. 2007 Oct 2;5(10):e261. doi: 10.1371/journal.pbio.0050261 (PMC1994990; doi:10.1371/journal.pbio.0050261)
Supplement: Supplementary file 8 [file pbio.0050261.st002.pdf]

Supplemental Table 2

	Number of Genes	Avg. Number of STREs	Avg. Position of nearest STRE
Genes Induced >2x at 13.5 hrs	10	3.4	-228.7
Genes Induced >2x at 18.5 hrs	38	2.736842105	-210.5526316
Genes Induced <2x at 18.5hrs	34	2.617647059	-279.3529412
	82	2.773809524	-241.4761905
