# Supplementary material for: MSN2 and MSN4 Link Calorie Restriction and TOR to Sirtuin-Mediated Lifespan Extension in Saccharomyces cerevisiae
Source: PLoS Biol. 2007 Oct 2;5(10):e261. doi: 10.1371/journal.pbio.0050261 (PMC1994990; doi:10.1371/journal.pbio.0050261)
Supplement: Supplementary file 9 [file pbio.0050261.st003.pdf]

Supplemental Table 3

Fig. 1a	average lifespan	wt 2%	wt CR	msn 2/4 2%	msn 2/4 CR
wt 2%	25		2.10E-03	9.68E-01	7.33E-01
wt CR	30.8			3.00E-04	5.00E-04
msn 2/4 2%	24.2				9.24E-01
msn 2/4 CR	23.9				

Fig. 1b	average lifespan	msn2	msn2 CR	msn4	msn4 CR
msn2	22.5		<1.0E-04	1.19E-01	<1.0E-04
msn2 CR	29.9			1.36E-02	7.83E-02
msn4	24.7				<1.0E-04
msn4 CR	33.2				

Fig. 1c	average lifespan	wt	wt + rap	msn 2/4	msn 2/4 + rap
wt	23.3		3.10E-02	7.72E-01	8.09E-01
wt + rap	26.9			5.52E-02	9.20E-03
msn 2/4	23.8				5.20E-01
msn 2/4 + rap	22.5				

Fig. 2d	average lifespan	wt	wt + rap	pnc1	pnc1 + rap
wt	22.3		1.60E-03	2.61E-01	6.55E-01
wt + rap	27.5			1.00E-04	2.00E-04
pnc1	20.9				5.13E-01
pnc1 + rap	21.4				

Fig. 2f	average lifespan	wt	wt PNC o/e	msn 2/4	msn 2/4 + PNC o/e
wt	23.2		4.40E-03	6.27E-01	2.42E-02
wt PNC o/e	28			1.20E-03	6.79E-01
msn 2/4	22				9.20E-03
msn 2/4 + PNC o/e	27.3				

Fig. 3e	average lifespan	wt 30	wt 37	msn 2/4 30	msn 2/4 37
wt 30	22.2		3.10E-03	2.09E-01	5.91E-02
wt 37	26.8			<1.0E-04	2.58E-01
msn 2/4 30	20.675				6.00E-04
msn 2/4 37	25.825				

Fig. S2c	average lifespan	sir2 fob1	sir2 fob1 + rap	sir2 hst2 fob1	sir2 hst2 fob1 + rap
sir2 fob1	25.7		4.79E-02	<1.0E-04	<1.0E-04
sir2 fob1 + rap	31.4			<1.0E-04	<1.0E-04
sir2 hst2 fob1	14.2				2.09E-01
sir2 hst2 fob1 + rap	15.6				
